# Molecular characterization and phylogenetic analysis of the complete genome of a porcine sapovirus from Chinese swine

**DOI:** 10.1186/1743-422X-6-216

**Published:** 2009-12-06

**Authors:** Shixing Yang, Wen Zhang, Quan Shen, Fen Huang, Yan Wang, Jianguo Zhu, Li Cui, Zhibiao Yang, Xiuguo Hua

**Affiliations:** 1 School of Agriculture and Biology, Shanghai Jiao Tong University, 800 Dong Chuan Road, Shanghai 200240, PR China; 2 School of Medical Science and Laboratory Medicine, Jiangsu University, 301 Xuefu Road, Zhenjiang, Jiangsu 212013, PR China

## Abstract

**Background:**

Porcine sapovirus was first identified in the United States in 1980, hitherto, several Asian countries have detected this virus. In 2008, the first outbreak of gastroenteritis in piglets caused by porcine sapovirus in China was reported. The complete genome of the identified SaV strain Ch-sw-sav1 was sequenced and analyzed to provide gene profile for this outbreak.

**Methods:**

The whole genome of Ch-sw-sav1 was amplified by RT-PCR and was sequenced. Sequence alignment of the complete genome or RNA dependent RNA polymerase (RdRp) gene was done. 3' end of ORF2 with 21-nt nucleotide insertion was further analyzed using software.

**Results:**

Sequence analysis indicated that the genome of Ch-sw-sav1 was 7541 nucleotide long with two ORFs, excluding the 17 nucleotides ploy (A) at the 3' end. Phylogenetic analysis based on part of RdRp gene of this strain showed that it was classified into subgroup GIII. Sequence alignment indicated that there was an inserted 21-nt long nucleotide sequence at the 3' end of ORF2. The insertion showed high antigenicity index comparing to other regions in ORF2.

**Conclusion:**

Ch-sw-sav1 shared similar genetic profile with an American PEC strain except the 21-nt nucleotide at the 3' end of ORF2. The insert sequence shared high identity with part gene of Sus scrofa clone RP44-484M10.

## Background

Caliciviridae is a family of positive sense single-stranded RNA viruses comprised of both human and animal pathogens [1]. Caliciviridae family contains four genera, Lagovirus, Vesivirus, Norovirus and Sapovirus [2]. Various caliciviruses possess common features. For example, they are small, non-enveloped virus, 27-38 nm in diameter. They possess a single-stranded, 7.3-8.3 kb plus-sense RNA genome, a single 56-71 kD capsid protein [3], and a polyprotein containing confering motifs of a putative 2C helicase, 3C-like protease, and 3D RdRp. SaV are recognized as emerging enteric pathogens in humans, swine and mink [4]. SaV infection may cause diarrhea especially in the younger [5]. It is currently divided into eight distinct genetic groups (GI-GVIII) based on the RdRp gene. Among these genetic groups, GIII can't infect humans but can be cultured *in vitro *in the presence of bile acid [6]. The genome of SaV consists of 7.1-7.5 kb nucleotide and encodes two or three open reading frames (ORFs). ORF1 encodes one polyprotein that contains coding sequences for the nonstructural proteins and the major capsid protein (VP1), ORF2 encodes the minor structural protein (VP2), while ORF3 is only present in strains from genotypes GI, GIV and GV, and encodes a small basic protein [7]. SaV is considered as a significant global enteropathogen of acute gastroenteritis [8]. Recently, it was shown that the host tropism of some calicivirus is less specific. Some calicivirus may have zoonotic potential, and animals such as domestic pig may be a reservoir for caliciviruses [9-11]. Porcine sapovirus was first identified in the United States by electron microscopy in 1980 [12] and genetically characterized as a sapovirus in 1999 [13]. Recently, SaV infections have been identified in Japan, South Korea, Venezuela, Hungary and Belgium [14-18]. In the United States, porcine sapovirus was also detected from Oyster [19]. Although porcine SaV was mainly detected in pigs, some studies indicated that some porcine SaV might be potential pathogencity transmitting to humans. For example, the porcine SaV strain (Sapovirus pig/43/06-18p3/06/ITA) isolated from Italy was most closely related to human SaV through the alignment of RdRp sequences, suggesting the possibility of a pig reservoir for human strains or vice versa [20]. We previously reported an outbreak of gastroenteritis in piglets in China caused by the first Chinese porcine SaV strain [21]. In this study, gene profile of this strain was investigated, the entire viral genome and 3' end of Ch-sw-sav1 were cloned and sequenced.

## Methods

### Samples

Porcine SaV positive fecal samples were collected from commercial pig farms in Shanghai as introduced in our previous study. Samples were converted to 20% (wt/vol) suspensions in phosphate-buffered saline (PBS) (0.01 M, pH 7.2 to 7.4) and clarified by centrifugation at 10,000 g for 10 min.

### Primers Design

In order to amplify the full-length sequence, 15 sets of primers were designed based on the sequences of AF18276 and DQ056363 that were previously submitted in the GenBank: Nucleotide sequence and position of the primers are listed in Table 1.

**Table 1:** Nucleotide sequences of the oligonucleotides used for PCR amplification and sequencing

Primer set	Primer name	Nucleotide sequence	Position
1	SP1F	GTGATCGGTGATGGCTAATTGCCG	1-14
	SP1R	TGGAGATGGTATCTGTCAGTGTG	645-667
2	SP2F	GGCAGTACATTTGTGAGGGGTG	543-564
	SP2R	CCTGTTCTGCTTTATCACCTCC	1170-1191
3	SP3F	GACGGTGGCTGCCATTAAAGCTG	1063-1085
	SP3R	GCAGTGTAGCCGCGTACTGAGC	1833-1854
4	SP4F	ATTGACGTGACAGCCCCCAC	1733-1752
	SP4R	TGTGGTTCTTGACTGGTGAG	2335-2354
5	SP5F	TGGTGGAGGCCTGTTCAGAGC	2223-2243
	SP5R	CCAAGTTGTGGGCTGTCAACAC	2757-2778
6	SP6F	CAGAGTCCTCCTGGTGGACATTC	2680-2702
	SP6R	ATTACCAAGCGCAACGCTAGGC	3340-3361
7	SP7F	CATGTGGCCAACATGTGTG	3243-3261
	SP7R	TGATTTGGTCAAGGTAGCC	3873-3891
8	SP8F	CCTTCTACAACACCAAATGATTGCC	3768-3792
	SP8R	AGGCCAGGATGTCAACACTGGCAC	4371-4394
9	SP9F	ATGTATGGATAGCCCTCAGATTG	4261-4283
	SP9R	GTCCACATCAACGGCCGCCGGCTCG	4890-4914
10	SP10F	AGCCAACAGACACTCCTGTGTTCC	4760-4783
	SP10R	CATGCCAGACCCTGATATTATCACC	5468-5492
11	SP11F	ACCTACACCAATGTCACCTGGAC	5328-5350
	SP11R	GTGCCACACCTACTATGACCACAG	5890-5913
12	SP12F	TCAAGCCTCCAAACCAAGCC	5784-5803
	SP12R	TGGCGGTCCATAAATGAGGTG	6395-6415
13	SP13F	TATGCAGCTTTGGCAATTCCC	6291-6311
	SP13R	TTGATCTTTAGCAACTGTATCTG	6892-6915
14	SP14F	TTGGATTGCAGGAGCAATGCAGG	6777-6799
	SP14R	TGTAAGGTTCGGTACGCGTAACC	7280-7303
15	SP15F1	TCAATTGGCTGGGTCACGTGAAG	7027-7049
	SP15F2	CAAACACCTTTGGTCCACCAAGG	7070-7092

### RNA extraction and cDNA synthesis

Viral RNA was extracted with TRIzol Reagent from supernatants of fecal suspensions, according to the manufacture's instructions. The cDNA synthesis was primed by Oligo dT_16 _or the reverse one of each set of primers using TaKaRa RNA PCR kit (TaKaRa, Japan) in a 10 μL reaction volume. The reaction condition was 40 min at 42°C, then 15 sec at 86°C.

### PCR and RACE amplifications of the full-length SaV genome

PCR was carried out in 50 μL reaction volume, containing 8 μL dNTP Mixture (25 mM), 5 μL 10×Ex-taq buffer, 0.2 μL Ex Taq, 1 μL (25 mM) of each primer, 10 μL of template and adding sterilize H_2_O to 50 μL. The reaction was done with the following profile: Activation of DNA polymerase at 95°C for 5 min, followed by 35 cycles of denaturation of DNA at 95°C for 40 sec, annealing at the 50°C for 1 min, extension at 72°C for 1 min and then followed by a final extension step at 72°C for 10 min. Purfied PCR products were ligated to pMD-18T vector (TaKaRa, Japan) and 3 to 5 positive colonies were sequenced.

### 3' RACE

The 3' RACE was carried out with TaKaRa RNA PCR Kit (TaKaRa, Japan) following the manufacture's instructions. Briefly, ten microliters of RNA were used as template to synthesize cDNA with AMV Reverse transcriptase for 1 h at 42°C. The external reverse primer which has a poly (T) tract was used to prime the cDNA synthesis. The cDNA was then amplified with the external forward primer (5'-TCAATTGGCTGGG TCACGTGAAG-3', nucleotide position numbers 7027-7049) and internal forward primer (5'- CAAACACCTTTGGTCCACCAAGG-3', nucleotide position numbers 7070-7092) with Ex Taq DNA polymerase (TaKaRa, Japan). The PCR reaction mixture was incubated for 2 min at 94°C, followed by 35 amplification cycles comprising denaturation at 94°C for 30 s, annealing at 65°C for 30 s, and extension at 72°C for 30 s. The product was extended for another 7 min at 72°C to ensure a full extension.

The PCR products were purified from 1% agarose gel using the QIAquick Gel Extraction kit (Qiagen, Gemany). Purified PCR products were ligated into pMD18-T Vector. For each product, three to five positive colonies were selected and sequenced.

### Phylogenetic analysis

Nucleotide sequences of the following calicivirus in Genbank were used in the phylogenic analysis (Table 2): SVs: Sapovirus Mc10/Japan (NC_010624), Sapovirus C12/Japan (AY603425), Sapovirus SaKaeo-15/Thailand (AY646855), Sapovirus Mc2/Japan (AY237419), Sapovirus Ehime1107/2002/JP(DQ058829), Sapovirus Mc114/Japan (AY237422), Sapovirus Hu/Dresden/pJG-Sap01/DE (AY694184), Sapovirus NongKhai-24/Thailand (AY646856), and Porcine enteric sapovirus/USA (AF182760); NVs: Norovirus mouse/Hannover1/2007/DEU (EU854589), Norwalk virus/USA (NC001959), Norwalk virus/Germany (AF093797), Norovirus Hu/GI/Otofuke/1979/JP (AB187514), Bovine calicivirus/UK (AJ011099), Bo/Dumfries/94/UK (AY126474), Human calicivirus strain Mc37/Japan (AY237415), Norwalk-like virus/Gifu'96/Japan (AB045603), Hawaii calicivirus/USA (HCU07611), Lordsdale virus (X86557), Norovirus Hu/GII-4/Hokkaido1/2006/JP (AB447427), Norovirus Hu/Houston/TCH186/2002/US (EU310927), Norovirus Hu/NLV/Oxford/B4S4/2002/UK (AY587986); VVs: FCV (M86379) and SMSV1 (SMU15301); LVs: RHDV (M67473) and EBHSV (Z69620). Sequencing reads from each PCR product were assembled using SeqMan II program (DNASTAR, Inc). Multiple sequence alignment was performed using CLUSTAL W method. The nucleotide identity and nucleotide divergence between complete Porcine SaV genomes was calculated using MegAlign program (DNASTAR, Inc). MEGA software was used to construct a phylogenetic tree, the reliability of the generated tree was evaluated by bootstrapping 1000 replicates. The same process was applied to analyse part of RNA dependent RNA polymerase genes, Nucleotide sequences of the following calicivirus in Genbank were used in the phylogenic analysis: Sapovirus Hu/Lyon/30338/98/F (AJ251991), Sapporo virus-Manchester (X86560), Sapporo virus-Houston/86 (U95643), Sapovirus Hu/Ehime/2K-814/2000 (AJ606698), Sapovirus Hu/Potsdam/2000/DEU (AF294739), Sapovirus Hu/Mex14917/2000 (AF435813), Sapovirus Hu/Hou7-1181 (AF435814), Sapovirus Hu/Ehime/99-1596/1999/JP (AJ606697), Sapovirus Hu/Ehime/01-1669/2001 (AJ606699), Sapovirus Hu/Arg39/1995/ARG (AY289803), Sapovirus pig/43/06-18p3/06/ITA (EU221477), Sapovirus Hu/Chiba/991172/1999 (AJ606691), Sapovirus Hu/cruise ship/2000/USA (AY289804), Sapovirus Hu/Bristol/1998/UK (AJ249939), Sapporo virus-London/29845 (U95645), Po/SaV/Giessen-08/2003/DE (EU122248), Po/SaV/Giessen-07/2004/DE (EU122246), Porcine enteric sapovirus swine/YiY1/2006/PRC (EU381231), Porcine enteric sapovirus/Venezuelan (DQ056363), Sapovirus swine/OH-JJ259/00/US (AY826423), Porcine enteric sapovirus/Japan (AB242875), Sapovirus swine/OH-MM280/03/US (AY823308), Sapovirus swine/NC-QW270/03/US (AY826426), PEC/swine-Id3/2005/HUN (DQ383274), Porcine enteric sapovirus/K8/JP (AB242873), Sapovirus Po/2053P4/Brazil (DQ359100), Sapovirus Po/OH-JJ681/2000/US (AY974192), Sapovirus Po/2014P2/Brazil (DQ359099), Sapovirus Po/OH-LL26/2002/US (AY974195), Porcine enteric sapovirus/K7/JP (AB221130). The sequence determined in current study was deposited in GenBank, the name was Ch-sw-sav1 and the accession number was FJ387164.

**Table 2:** Summary of sapovirus strains and representative strains for Lagovirus, Vesivirus, and Norovirus genera and NB-like viruses used in sequence analysis

Strains	Genus/genogroup	GenBank accession no.
Sapovirus Mc10/Japan	SaV/GII	NC_010624
Sapovirus C12/Japan	SaV/GII	AY603425
Sapovirus SaKaeo-15/Thailand	SaV/GII	AY646855
Sapovirus Mc2/Japan	SaV/GII	AY237419
Sapovirus Ehime1107/2002/JP	SaV/GII	DQ058829
Sapovirus Mc114/Japan	SaV/GI	AY237422
Sapovirus Hu/Dresden/pJG-Sap01/DE	SaV/GI	AY694184
Sapovirus NongKhai-24/Thailand	SaV/GV	AY646856
Porcine enteric sapovirus/USA	SaV/GIII	AF182760
Norovirus mouse/Hannover1/2007/DEU	Mouse NoV	EU854589
Norwalk virus/USA	NoV/GI	NC001959
Norwalk virus/Germany	NoV/GI	AF093797
Norovirus Hu/GI/Otofuke/1979/JP	NoV/GI	AB187514
Bovine calicivirus/UK	Bovine calicivirus	AJ011099
Bo/Dumfries/94/UK	Bovine calicivirus	AY126474
Human calicivirus strain Mc37/Japan	NoV/GII	AY237415
Norwalk-like virus/Gifu'96/Japan	NoV/GII	AB045603
Hawaii calicivirus/USA	NoV/GII	HCU07611
Lordsdale virus	NoV/GII	X86557
Norovirus Hu/GII-4/Hokkaido1/2006/JP	NoV/GII	AB447427
Norovirus Hu/Houston/TCH186/2002/US	NoV/GII	EU310927
Norovirus Hu/NLV/Oxford/B4S4/2002/UK	NoV/GII	AY587986
Feline calicivirus	FCV	M86379
San Miguel sea lion virus serotype 1	SMSV1	SMU15301
European brown hare syndrome virus	RHDV	M67473
European brown hare syndrome virus	EBHSV	Z69620

### 3' end of ORF2 partial sequences analysis

Six available Porcine SaVs partial sequences of 3' end of ORF2 were retrieved from GenBank, according to sequence alignment. As follows: OH-MM-280-03-US (AY823308), PEC-USA (AF182760), strain LL14 (AY425671), OH-JJ-259-00-US (AY826423), NC-QW-270-03-US (AY826426). Nucleotide sequence and protein were aligned by CLUSTAL W method using DNAstar software, antigen index was analysed by protean using DNAstar software.

## Results

### Genomic organization of Ch-sw-sav1 virus

**Figure 1:**
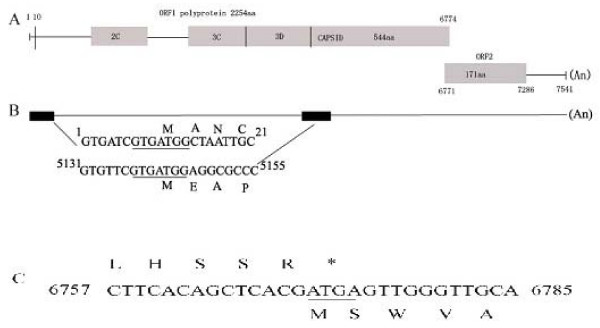
**Genomic characteristic of Ch-sw-sav1**. A. Schematic of the genomic organization of Ch-sw-sav1 showing the two predicted ORFs: ORF1, encoding a polyprotein fused to and contiguous with the capsid protein (VP1), forming a large polyprotein; and ORF2 encoding a small basic protein (VP2) of unknown function. B. Schematic of the conserved nucleotide sequence motifs at the 5' termini of the genomic and predicted subgenomic RNAs. The Kozak context, favorable for translation initiation, is underlined. C. Aligned nucleotide and predicted amino acid sequences at the junction between ORF1 and ORF2. ORF2 overlaps the 3' end of ORF1 by 4nt (underlined).

The complete RNA genome of Ch-sw-sav1 is consisted of 7541 nt, excluding its 3' end poly(A) tail, was longer than the USA strain (GenBank no.: AF182760). It's A, C, G, U ribonucleotide composition was 19%, 14.3%, 33.3%, and 33.3%, respectively. The 5' terminus genomic RNA started with the featured trinucleotide GTG. Similar to the genomes of SVs and LVs, the Ch-sw-sav1 genome contained two predicted ORFs. ORF1 was 6765 bases (2255 aa) in length encoding non-structural proteins and VP1 (544aa). ORF2, consisting of 516 bases (nt 6771-7286), was predicted to encode VP2 protein with 172 aa. (Fig. 1A). The predicted polyprotein encoded by ORF1 contained the common 2C helicase (GPPGIGKT), 3C protease (GDCG), and RdRp (GLPSG and YGDD) motifs that were highly conserved in all calicivirus. The PPG motif was also present in the predicted VP1 (data not shown).

### Sequence comparison

**Figure 2:**
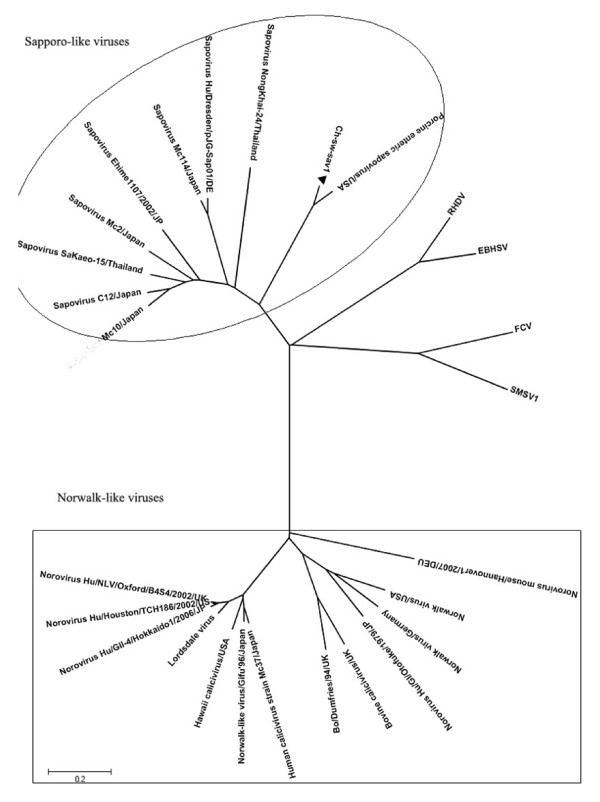
**Phylogenetic tree generated for the sequences in the complete genome**. Phylogenetic tree constructed on the basis of the complete genome sequence. All sequences were collected from GenBank. The virus detected in this study was marked with black triangle. Trees were prepared using the Treeview programs and all branches supported based on 100 bootstrapped data sets.

**Figure 3:**
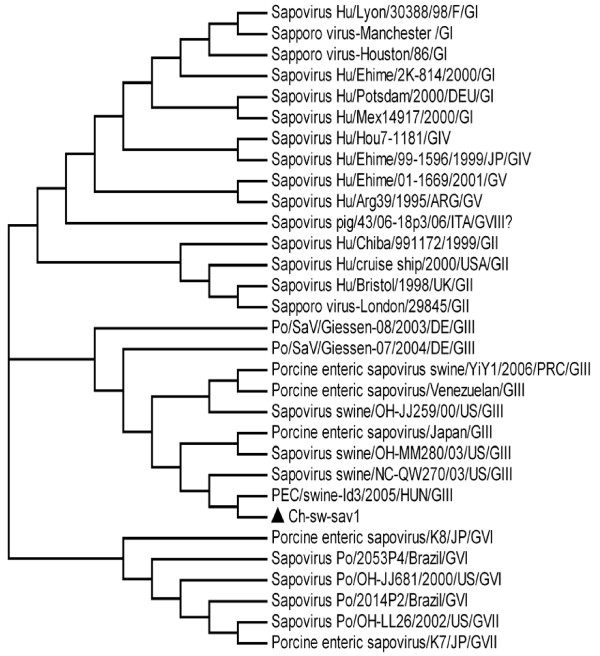
**Unrooted phylogenetic tree of calicivirus RdRp gene sequences constructed by the neighbor-joining method**. Phylogenetic tree constructed on the basis of concentrated RdRp gene sequence. Trees were prepared using the Treeview programs and are based on 100 bootstrapped data sets. All sequence used in this analysis were collected from GenBank. The virus detected in this study was marked with black triangle and it was composed of a cluster with PEC/swine-Id3/2005/HUN and Sapovirus swine/NC- QW270/03/US, they also belong to porcine SaV genotype GIII.

We compared the entire genome sequence identities of Ch-sw-sav1 with those of other calicivirus, A phylogenetic tree based on the entire genome sequence showed that Ch-sw-sav1 was closely related to the SLVs than to the other caliciviruses (Fig. 2). The phylogenetic tree was then constructed on the basis of concentrated alignments of RNA dependent RNA polymerase gene sequence of 31 SaV strains by the neighbour-joining method (Fig. 3). All eight genotypes were separated into corresponding lineages. Within the genotype-3 lineage, there were four distinct subgroups. The analysis indicated that Ch-sw-sav1 formed a subgroup together with two USA strains, one Japanese strain and one Hungary strain. Further analysis indicated Ch-sw-sav1 shared 82.2%-91.2% identities with the other GIII SaV strains, and it was closely related to the Hungary variant DQ383274 (Table 3). Whereas, it was less similar (< 57.1%) to the strains of GI, GII, GIV, GV, GVI, GVII, GVIII.

**Table 3:** Percentages of nucleotide sequence identity of Ch-sw-sav1 with other caliciviruses in regions aligned for phylogeny

Strain	Genogroupe	**GenBank accession no**.	% Identity
Hu/Lyon/30338/98/F	GI	AJ251991	47.7
Sapporo virus-Manchester	GI	X86560	42.7
Sapporo virus-Houston/86	GI	U95643	48.5
Hu/Ehime/2K-814/2000	GI	AJ606698	40.3
Hu/Potsdam/2000/DEU	GI	AF294739	57.1
Hu/Mex14917/2000	GI	AF435813	40.4
Hu/Hou7-1181	GIV	AF435814	50.3
Hu/Ehime/99-1596/1999/JP	GIV	AJ606697	45.5
Hu/Ehime/01-1669/2001	GV	AJ606699	43.8
Hu/Arg39/1995/ARG	GV	AY289803	42.1
pig/43/06-18p3/06/ITA	GVIII?	EU221477	29.0
Hu/Chiba/991172/1999	GII	AJ606691	40.0
Hu/cruise ship/2000/USA	GII	AY289804	22.9
Hu/Bristol/1998/UK	GII	AJ249939	43.2
Sapporo virus-London/29845	GII	U95645	47.2
Po/SaV/Giessen-08/2003/DE	GIII	EU122248	88.4
Po/SaV/Giessen-07/2004/DE	GIII	EU122246	86.1
swine/YiY1/2006/PRC	GIII	EU381231	84.2
Porcine sapovirus/Venezuelan	GIII	DQ056363	86.1
swine/OH-JJ259/00/US	GIII	AY826423	86.1
Porcine enteric sapovirus/Japan	GIII	AB242875	84.5
swine/OH-MM280/03/US	GIII	AY823308	82.2
swine/NC-QW270/03/US	GIII	AY826426	86.7
PEC/swine-Id3/2005/HUN	GIII	DQ383274	91.2
Porcine enteric sapovirus/K8/JP	GVI	AB242873	20.2
Po/2053P4/Brazil	GVI	DQ359100	18.6
Po/OH-JJ681/2000/US	GVI	AY974192	28.3
Po/2014P2/Brazil	GVI	DQ359099	16.3
Po/OH-LL26/2002/US	GVII	AY974195	29.2
Porcine enteric sapovirus/K7/JP	GVII	AB221130	18.4

**Figure 4:**
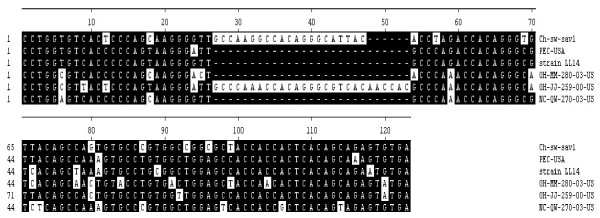
**Nucleotide acid alignment of 3' end sequences of VP2 among six porcine SaV strains**. The numbers above the alignment show the nucleotide location in the ORF2. The nucleotide with the white background is differential. The inserted sequence of Ch-sw-sav1 is from 27-nt to 46-nt

**Figure 5:**
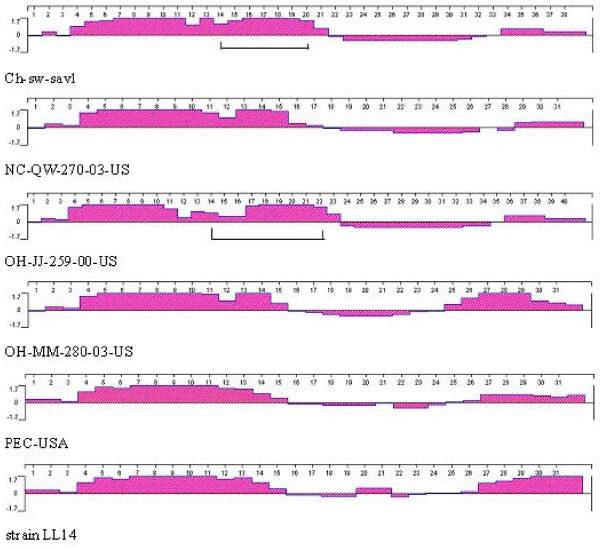
**Antigen index analysis of 3' end sequences of VP2 among six porcine SaV strains**. Antigen index is analysed by protean using DNAstar software. The regions marked by scale are the site of inserted sequence.

The 5' terminus of the genomic and predicted subgenomic RNAs of Ch-sw-sav1 possessed leader sequences with a Kozak structure (G/ANNATGG), which was favourable for translation initiation of eukaryotic mRNA [22] (Fig. 1B), similar to that of PEC (GenBank No.: AF182760) [13], The VP1 region (544aa) of Ch-sw-sav1 was the same in length as in PEC and slightly shorter than those of SaVs of human origin. The ORF2 overlapped 4 nucleotides with VP1 gene, common to others in PEC (Fig. 1C), but the length of ORF2 was distinct. Sequence alignment based on the 3' end of ORF2 of six available sequences in GenBank indicated that there was 21-nt long nucleotide sequence insertion, which was similar to the gene module of OH-JJ-259-00-US strain (GenBank No.: AY826423) with 27-nt long nucleotides inserted (Fig. 4). Analysis of antigen index showed that the inserted sequence was within the affluent antigen site besides another at the 3' end of ORF2 (Fig. 5).

## Discussion

Sapporo virus was identified in 1982 from an outbreak of diarrhea in an orphanage in Sapporo, Japan [23]. Schuffenecker [24] classified them into three major genetic groups. Furthermore, it has been divided into eight genogroups based on the genetic diversity of the viral polymerase [25]. PEC, the first of pig origin, was discovered in 1980s in the United States and belongs to SaV GIII [12]. Hitherto, SaV has been identified in many countries [14-18]. Traditionally, we thought only SaV GIII infected pig. However, strains detected in USA and Italy that belonged to new genotype showed high homology with human SaVs respectively. It indicated that animals might act as reservoirs for human caliciviruses. So it is necessary to analyze the genetic profile of porcine SaV for the first step of controlling the pathogen. In February 2008, we reported the first outbreak of gastroenteritis caused by porcine SaV in piglets in China mainland. It may be caused by simultaneous contact with virus polluted water or food and the virus gene profile was further investegated. Ch-sw-sav1 was chosen to be sequenced and compared with other SaV published. Results showed that it shared high homology with PEC for the similar gene structure and similar sequence motif at 5' terminus that was favorable for translation initiation of eukaryotic sequence [22]. However, there was 21-nt nucleotide insertion at the 3' end of ORF2 of Ch-sw-sav1. The inserted sequence had a high antigenicity index analyzed with DNAstar software. It's predicted that ORF2 encodes capsid protein that is correlative with the assembly, antigenicity and receptor interations of SaV. So the inserted sequence may affect antigenicity profile or other profiles of capsid protein which need to be further identified [1]. Accordingly, in phylogenetic analysis, we classified Ch-sw-sav1 into Genogroup III of SaV basing on the partial RdRp gene sequence, and it shared highest nucleotide identity with the Hungary SaV (91.2%) which was isolated from a diarrheaed pig [17].

The porcine SaV strain in the present study came from an outbreak of gastroenteritis in piglets group, which had inserted sequence at the 3' end of ORF2. The role of the inserted sequence was unknown, but it is highly divergent in sequence and differs in size in caliciviruse *s*. Since the ORF2 protein is functionally conserved and may be involved in protein-protein interactions or protein-nucleic acid interactions during replication based on its strong positive charge. The inserted sequence likely has special biological function. So establishing full-length infectious clones containing or not containing this inserted fragment would now be the next step towards the identification of this fragment involved in symptomatology and pathogenicity.

## Conclusion

Complete sequence of the first Chinese porcine SaV was determined and analyzed providing a gene profile of porcine SaV presented in swine population in China today. Sequence analysis showed that it was classified into genogroup III with two ORFs. A 21-nt insertion in ORF2 changed antigenicity index of capsid protein.

## Competing interests

The authors declare that they have no competing interests.

## Authors' contributions

All authors participated in the planning of the project. XH was the leader of the project. SY and WZ amplified the complete genome and analyzed the genome profile. QS and FH went on the sequence alignment. All authors read and approved the final manuscript.
